# Childhood temperamental, emotional, and behavioral characteristics associated with mood and anxiety disorder s in adolescence: A prospective study

**DOI:** 10.1111/acps.13522

**Published:** 2022-11-25

**Authors:** Nora R. Bakken, Laurie J. Hannigan, Alexey Shadrin, Guy Hindley, Helga Ask, Ted Reichborn‐Kjennerud, Martin Tesli, Ole A. Andreassen, Alexandra Havdahl

**Affiliations:** ^1^ NORMENT Centre, Institute of Clinical Medicine, University of Oslo and Division of Mental Health and Addiction Oslo University Hospital Oslo Norway; ^2^ Nic Waals Institute Lovisenberg Diaconal Hospital Oslo Norway; ^3^ Department of Mental Disorders Norwegian Institute of Public Health Oslo Norway; ^4^ Population Health Sciences, Bristol Medical School University of Bristol Bristol UK; ^5^ Institute of Psychiatry, Psychology and Neuroscience King's College London London UK; ^6^ Department of Psychology University of Oslo Oslo Norway; ^7^ Institute of Clinical Medicine University of Oslo Oslo Norway; ^8^ KG Jebsen Centre for Neurodevelopmental Disorders University of Oslo Oslo Norway

**Keywords:** anxiety disorders, depressive disorders, development, irritability, MoBa

## Abstract

**Background:**

Mood and anxiety disorders account for a large share of the global burden of disability. Some studies suggest that early signs may emerge already in childhood. However, there is a lack of well‐powered, prospective studies investigating how and when childhood mental traits and trajectories relate to adolescent mood and anxiety disorders.

**Methods:**

We here examine cross‐sectional and longitudinal association between maternally reported temperamental traits, emotional and behavioral problems in childhood (0.5–8 years) and clinical diagnosis of mood or anxiety (“emotional”) disorders in adolescence (10–18 years), using the prospective Norwegian Mother, Father and Child Cohort Study (MoBa) of 110,367 children.

**Results:**

Logistic regression analyses showed consistent and increasing associations between childhood negative emotionality, behavioral and emotional problems and adolescent diagnosis of emotional disorders, present from 6 months of age (negative emotionality). Latent profile analysis incorporating latent growth models identified five developmental profiles of emotional and behavioral problems. A profile of early increasing behavioral and emotional problems with combined symptoms at 8 years (1.3% of sample) was the profile most strongly associated with emotional disorders in adolescence (OR vs. reference: 5.00, 95% CI: 3.70–6.30).

**Conclusions:**

We found a consistent and increasing association between negative emotionality, behavioral and emotional problems in early to middle childhood and mood and anxiety disorders in adolescence. A developmental profile coherent with early and increasing disruptive mood dysregulation across childhood was the profile strongest associated with adolescent emotional disorders. Our results highlight the importance of early emotional dysregulation and childhood as a formative period in the development of adolescent mood and anxiety disorders, supporting potential for prevention and early intervention initiatives.

**Significant outcomes**

- This prospective study indicates that childhood negative emotionality, behavioral and emotional difficulties are consistently and increasingly associated with adolescent diagnosis of anxiety and depression, with differences present already from 6 months of age (negative emotionality).
- We identified distinctive trajectories of childhood emotional and behavioral problems, finding a combined symptom profile with high and increasing childhood symptoms to be the profile strongest related to adolescent diagnosis.
- Collectively our findings highlight the importance of childhood as a formative period in development of adolescent mood and anxiety disorders, emphasizing emotional dysregulation as a potential important trait and supporting potential for further research investigating prevention and early intervention initiatives.

**Limitations**

- The childhood traits investigated in this study are based on questionnaires filled out by the mother, that is, the traits investigated does not only reflect characteristics of the child, but also the maternal perception of their children.
- MoBa is a population‐based pregnancy cohort, thus despite using clinical diagnoses as outcome, potential selection bias in severity of diagnoses can limit generalizability of our results to the full range of individuals seen in clinical settings.

## 1 INTRODUCTION

Mood and anxiety disorders are leading global causes of disability. According to the Global Burden of Disease study from 2019, 29 million and 47 million disability‐adjusted life years (DALYs) were lost due to anxiety and depressive disorders respectively, accounting for the highest relative burden in children and adolescents.^1^ The disorders tend to present with a chronic and recurrent course, where co‐occurrence with other mental disorders and continuity into adulthood is common.^2^, ^3^ Early identification and treatment have been shown to significantly improve prognosis,^4^ leading to calls for progress in prevention strategies for mood and anxiety disorders.^5^, ^6^

Childhood and adolescence have been described as an optimal time for prevention and early intervention, targeting mental health problems in general, and anxiety and depression spesifically.^5^, ^7^ The period of childhood through adolescence is a risk phase for the first occurrence of symptoms of anxiety and depression.^2^, ^8^ Furthermore, childhood and adolescence are critical periods for brain development,^9^ as well as psychosocial adaptations.^10^ However, detecting early stages of mood and anxiety disorders have proven difficult, especially in children and adolescents where symptoms tend to be nonspecific, developmentally dependent, and with greater individual variability.^11^, ^12^

Temperamental traits and internalizing and externalizing behaviors influence risk of mood and anxiety disorders in children.^13^, ^14^, ^15^ Neuroticism and behavioral inhibition have, despite variations in effect sizes, shown a consistent association with emotional disorders.^16^, ^17^ However, the relationship between childhood internalizing and externalizing problems and later mental disorders, are unclear.^18^ The evidence suggest that mental health problems in childhood increase risk of adult mental disorders,^18^ in line with previous prospective and retrospective studies.^19^ However, the heterogeneity in findings are large, calling for more comprehensive assessments and large, prospective studies.

Longitudinal studies in child and adolescent populations suggest that children and adolescents differ in developmental trajectories of symptoms of depression,^20^ anxiety^21^ and behavioral problems.^22^ However, there is a lack of knowledge about precursors to later mood and anxiety disorders that are observable in the earliest years of life (<4 years), as well as the relative importance of temperamental traits and symptoms of mental disorders for later diagnostic outcomes.^20^, ^21^ To enable development of prevention and early intervention efforts, better knowledge about the early signs and developmental trajectories associated with mood and anxiety disorders is needed.^5^

Here we leverage a prospective population‐based pregnancy cohort, the Norwegian Mother, Father and Child Cohort Study (MoBa) of 114,326 children to examine when and how temperamental traits and emotional and behavioral problems in infancy to middle childhood (0.5 to 8 years of age) associate with mood and anxiety disorders in adolescence (10–18 years of age). We characterize trajectories of emotional and behavioral problems during childhood and assess their relationships to mood and anxiety disorders in adolescence.

## 2 MATERIALS AND METHODS

### 2.1 Study population

MoBa is a population‐based pregnancy cohort study conducted by the Norwegian Institute of Public Health. Participants were recruited from all over Norway from 1999 to 2008. The women consented to participation in 41% of the pregnancies.^23^, ^24^ The current study is based on version 12 of the quality‐assured maternally filled‐out questionnaires released for research in January 2019. The study sample included all children in MoBa with data on at least one of the five assessment waves between age 6 months and 8 years and linkage data from the Norwegian Patient Registry (NPR) in adolescence (age 10–18 years) (*n* = 110,367). A participant flow chart is presented in Supplementary Figure S1.

The establishment of the MoBa cohort and initial data collection was based on a license from the Norwegian Data Protection Agency and approval from The Regional Committees for Medical and Health Research Ethics, and the MoBa cohort is now following the regulations of the Norwegian Health Registry Act. The current study was approved by the administrative board of the Norwegian Mother, Father and Child Cohort Study led by the Norwegian Institute of Public Health and The Regional Committees for Medical and Health Research Ethics (2016/1226/REK sør‐øst C) in Norway.

### 2.2 Measures

#### 2.2.1 Temperament and personality

Temperament and personality measures were based on questionnaires filled out by the mother. Infant temperament was measured at 6 months of age using questions from the Infant Characteristics Questionnaire (ICQ).^25^ Two dimensions were evaluated on a 7‐point Likert scale: 7 items from the fussy/difficult subscale (negative emotionality) and 2 items representing positive emotionality. Early childhood temperament was measured by the four dimensions of Emotionality, Activity and Shyness Temperament Questionnaire (EAS) when the child was 18 months, 3 years and 5 years.^26^ Emotionality (negative emotionality), shyness, sociability and activity were measured by 3 items each. At 8 years, personality was evaluated by 6 items from each of the five dimensions of the Short Norwegian Hierarchical Personality Inventory for Children (NHiPIC‐30): Neuroticism (negative emotionality), extraversion, benevolence (agreeableness), imagination and conscientiousness.^27^ Due to theoretical continuity,^28^ overlapping phenotypes and high‐correlation between fussy temperament (ICQ), emotionality (EAS), and neuroticism (NHiPIC‐30) we have interpreted the measures as a common continuum of negative emotionality (See Figures S2 and S3).

#### 2.2.2 Emotional and behavioral problems

Emotional and behavioral problem measures were based on questionnaires filled out by the mother. The Child Behavior Checklist (CBCL) was used to measure emotional problems (internalizing items) and behavioral problems (externalizing items) at 18 months, 3 years and 5 years.^29^ More specific measures of symptoms of emotional and behavioral disorders were used at 8 years. A 13‐item Short Mood and Feelings Questionnaire (SMFQ) measured depressive symptoms.^30^ Symptoms of anxiety were measured on a 5‐item short form of Screen for Child Anxiety Related Disorders (SCARED).^31^ Symptoms of conduct problems (8 items), oppositional defiant problems (8 items), hyperactivity (9 items) and inattention (9 items) were assessed by the Rating Scale for Disruptive Behavior Disorders (RS‐DBD).^32^

An overview of individual items included in each measure is presented in Table S1. Ordinal Cronbach alpha for each scale is presented in Table S2.

#### 2.2.3 Emotional (mood or anxiety) disorder present in adolescence (10–18 years of age)

Information on diagnoses in adolescence was retrieved from the Norwegian Patient Registry comprising International Classification of Diseases Tenth Revision (ICD‐10)^33^ diagnoses registered in specialist health care services from 2008 through 2018. As anxiety and mood disorders commonly co‐occur and have overlapping symptomatology, we examined the disorders as a combined category of emotional disorders optimizing utility for the purpose of prevention and early detection.^34^ Emotional disorders comprised mood disorders including depressive and bipolar disorders (F30‐F39), anxiety disorders (F40‐F41) and emotional disorders with onset usually occurring in childhood and adolescence (F92‐F93) received from 10 years of age. We also defined non‐overlapping subcategories of depressive disorders (F32‐F34.1) and anxiety disorders (F40‐F41 or F930, F931 or F932).

#### 2.2.4 Covariates

Given known sex differences in the prevalence of emotional disorders and the wide range in birth years in MoBa, we included sex and birth year as covariates in all analyses. Information on participants' sex and birth year were retrieved from the Medical Birth Registry of Norway (MBRN), a national health registry containing information about all births in Norway.

### 2.3 Statistical analysis

Associations between maternally reported measures of temperament/personality, emotional and behavioral problems in childhood (6 months–8 years) and clinical diagnosis of emotional disorder in adolescence (10–18 years) were evaluated in separate logistic regression analyses. All measures were standardized with a mean of 0 and SD of 1 prior to analysis to improve comparability. To account for dependency in the data resulting from some mothers reporting on several children (siblings), all logistic regression analyses were calculated with Huber‐White robust standard errors clustered by maternal identity.^35^, ^36^ We applied a 5% alpha level and used False discovery rate (Benjamini–Hochberg) correction to account for multiple testing.^37^ Missing values were handled by case‐wise deletion. To evaluate if our results were driven by mood or anxiety disorders, we conducted sensitivity analyses stratifying for the two main categories of emotional disorders.

Latent profile analysis incorporating latent growth models for longitudinally measured traits were used to evaluate how developmental trajectories of emotional and behavioral problems in childhood relate to diagnosis of emotional disorder in adolescence. Parallel process latent growth models were applied to the CBCL domains emotional and behavioral problems, then measures of more specific symptom domains from the 8‐year questionnaires (SCARED, SMFQ, and RS‐DBD) were incorporated alongside the growth processes in latent profile analysis to classify the developmental profiles (for further details see^38^ and Figure S4). A manual 3‐step maximum likelihood approach using Mplus was applied for profile generation and prediction of distal outcome.^39^, ^40^ Sibling relatedness was accounted for by clustering within mothers. Missing data were handled by full information maximum likelihood. Model selection was, in accordance with other developmental studies, determined by classical fit statistics, entropy, the substantive interpretability of each profile, and the proportion of individuals assigned to the smallest profile (we used a threshold of >1%).^41^

R‐version 4.0.3 was used for all analyses except parts of the developmental models utilizing Mplus version 8.3.^42^ The R‐package “Phenotools” (https://github.com/psychgen/phenotools), was used to calculate scores for the scales and prepare diagnostic data. For further details on data analyses see supplementary text 1.

#### 2.3.1 Sensitivity analyses

To evaluate whether our results were affected by the age of onset of disorder, we repeated the analyses restricting the outcome to adolescent‐onset emotional disorder (*n* = 2878) and childhood‐onset adolescent‐persistent emotional disorder (*n* = 461) defined as first registered diagnoses after or before the age of 10 years respectively. Main analyses were also stratified for sex, assessing validity for both males and females.

#### 2.3.2 Consideration of missing data and attrition

We examined whether response rates for childhood measures differed between children with and without adolescent diagnosis of emotional disorder (See Table S3). We also examined characteristics of individuals excluded from developmental profile analyses due to missing values in all relevant variables (n = 28,480) (See Table S4).

## 3 RESULTS

Demographic information and descriptive information on key study variables are presented in Table 1 and Table S5.

**TABLE 1:** Demographic information for the study sample

	Emotional disorder (*n* = 3339)	No emotional disorder (*n* = 107,028)	Total (*n* = 110,367)
Sex, male, *n* (%)	1598 (47.86%)	54,912 (51.31%)	56,510 (51.20%)
Age end of follow‐up, years, *m* (SD)	14.28 (2.11)	13.00 (2.12)	13.04 (2.13)
Age first emotional disorder, years, *m* (SD)	12.13 (2.63)	–	12.13 (2.63)
Highly educated mother or father, *n* (%)	1684 (61.37%)	66,406 (72.83%)	68,090 (72.49%)
Marital status, *n* (%)^a^	3102 (92.90%)	102,355 (95.63%)	105,457 (95.55%)
Maternal age, *m* (SD)^b^	29.37 (4.92)	30.14 (4.63)	30.12 (4.64)
Parity, *n* (%) primiparous	1516 (45.40%)	46,823 (43.75%)	48,339 (43.80%)
Mother's country of birth			
Norway, *n* (%)	3063 (93.56%)	96,519 (91.82%)	99,582 (91.87%)
Other high‐income‐country, *n* (%)	139 (4.25%)	4798 (4.56%)	4937 (4.55%)
Other GDB 7 super region country, *n* (%)	72 (2.20%)	3804 (3.62%)	3876 (3.58%)
Co‐occurring diagnoses			
OCD, *n* (%)	116 (3.47%)	345 (0.32%)	461 (0.42%)
ADHD, *n* (%)	613 (18.36%)	3936 (3.68%)	4549 (4.12%)
CD, *n* (%)	101 (3.02%)	487 (0.46%)	588 (0.53%)

Abbreviations: ADHD, attention‐deficit/hyperactivity disorder; CD, conduct disorders; co‐occurring diagnoses, additional diagnosis during follow‐up; OCD, obsessive–compulsive disorder.

^a^ *n* (%) married/registered partner/co‐habitant.

^b^ Mean maternal age at pregnancy is calculated for mothers between age 17 and 45 due to lack of information on age for mothers above or below this age‐range (*n* = 58).

Of the 110,367 included individuals, 3339 were registered with a diagnosis of emotional disorder during follow‐up, stratified into the following sub‐categories: depressive disorder

(*n* = 644), anxiety disorder (*n* = 1423), depressive and anxiety disorder (*n* = 259) and other emotional disorders, including bipolar disorder (*n* = 1013).

### 3.1 Individual childhood trait measures associations with emotional disorder present in adolescence

**FIGURE 1:**
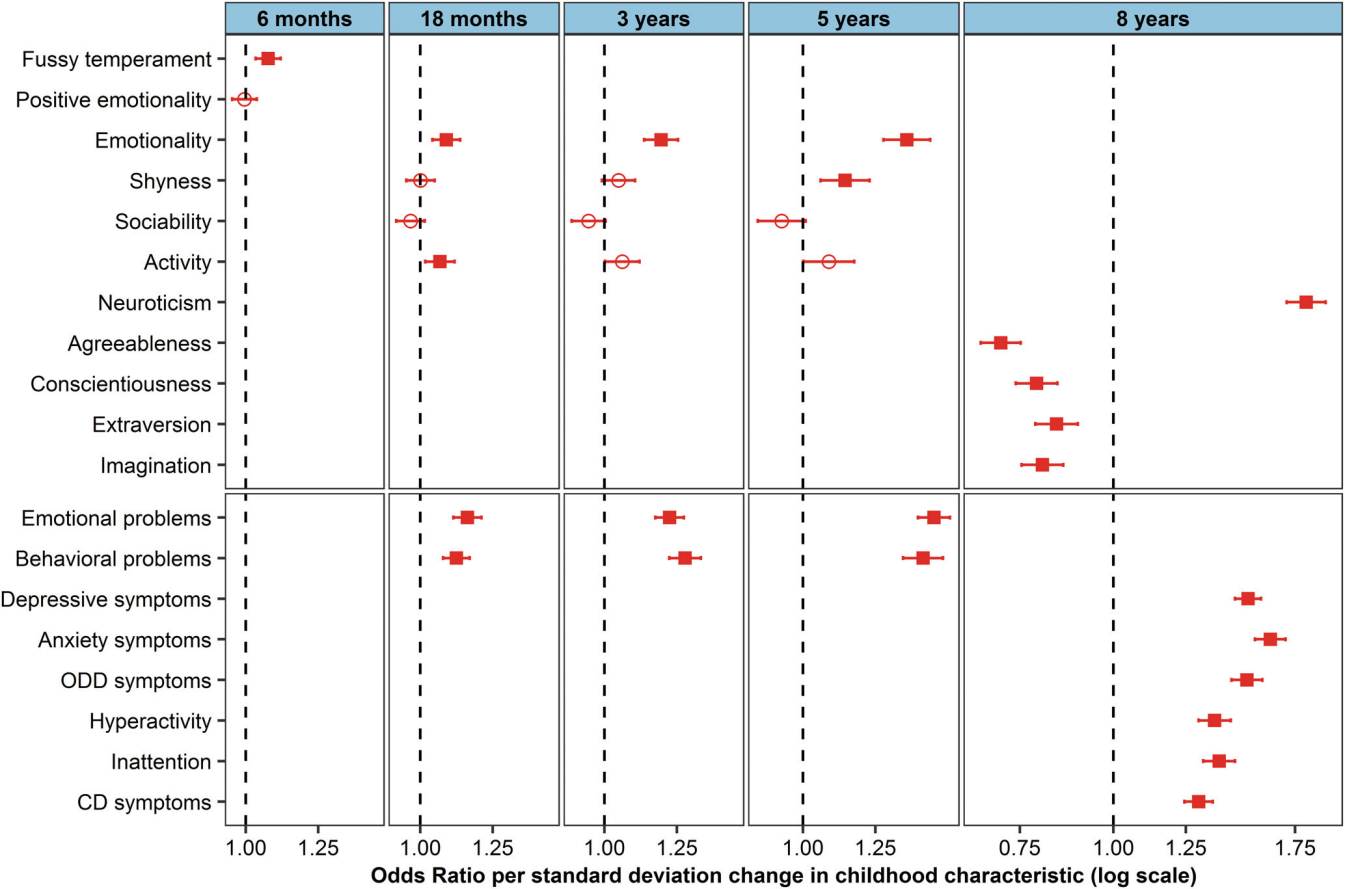
Association between temperamental traits and mental health problems in childhood and any emotional disorder in adolescence. Odds ratio for the difference in odds of any emotional disorder in adolescence (10–18 years) per standard deviation change in score for temperamental traits and mental health problems in childhood (6 months–8 years). Circle shaped point‐estimate is non‐significant after false discovery rate (Benjamini‐Hochberg) correction, square shaped point‐estimate is significant with *p*‐value <0.05 after false discovery rate (Benjamini–Hochberg) correction.

Results from logistic regression analysis indicated positive associations between emotional disorders in adolescence and traits of negative emotionality at 6 months, 18 months, 3 years, 5 years and 8 years (all *p*‐values^FDR^ <0.001), as well as shyness at 5 years (*p*‐value^FDR^ 0.001) and activity at 18 months (*p*‐value^FDR^ 0.011). Emotional and behavioral problems measured at 18 months, 3 years, 5 years and 8 years of age were consistently and with increasing effect sizes associated with emotional disorder in adolescence (all *p*‐values^FDR^ <0.001). Negative associations with emotional disorders were found for agreeableness, conscientiousness, imagination, and extraversion (all *p*‐values^FDR^ <0.001). Results are presented in Figure 1 and Table S6.

**FIGURE 2:**
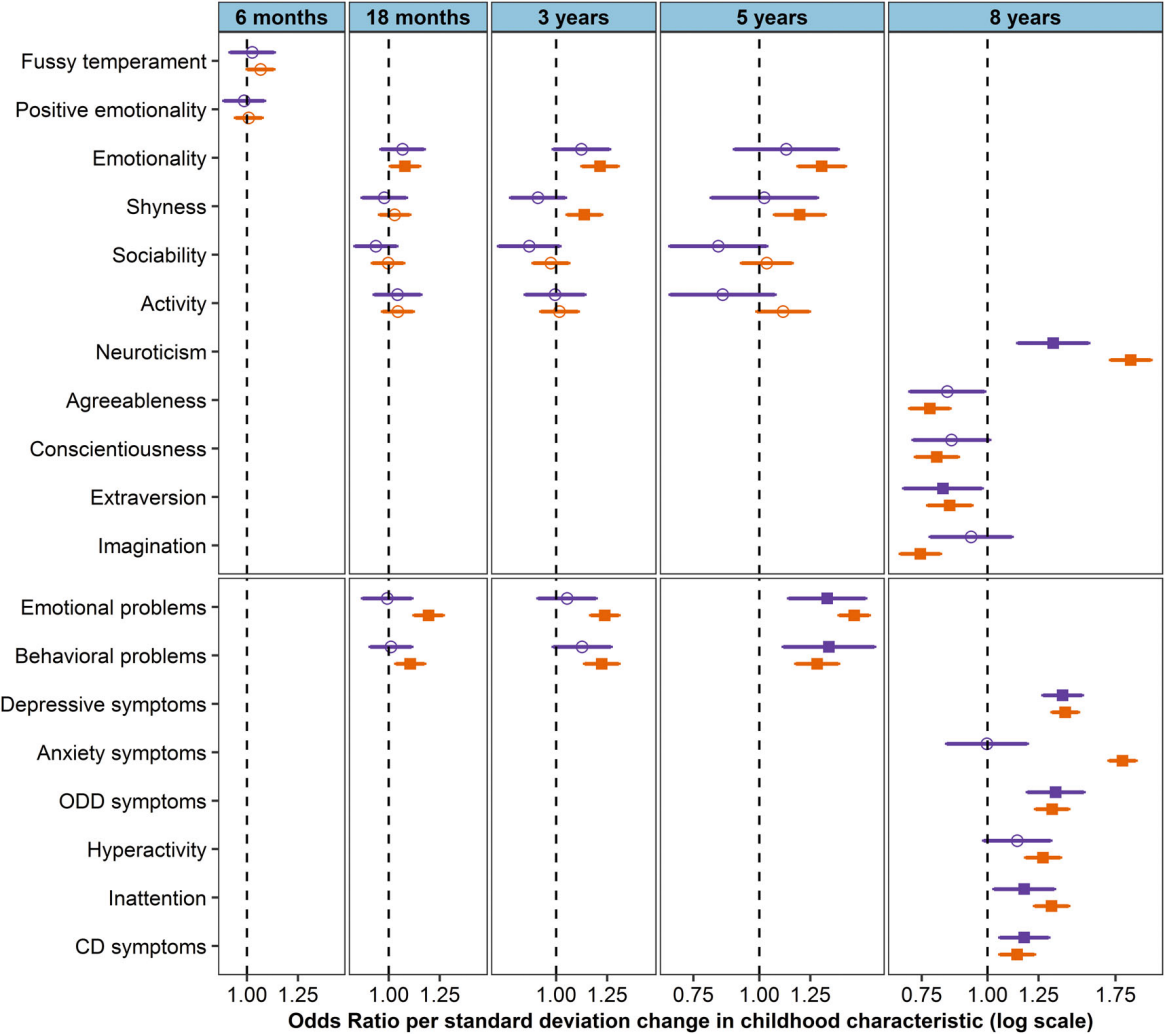
Association between temperamental traits and mental health problems in childhood and any anxiety or depressive disorder in adolescence. Odds ratio for the difference in odds of depressive disorder (purple) or anxiety disorder (orange) in adolescence (10–18 years) per standard deviation change in score for temperamental traits and mental health problems in childhood (6 months–8 years). Circle shaped point‐estimate is non‐significant after false discovery rate (Benjamini–Hochberg) correction, square shaped point‐estimate is significant with *p*‐value <0.05 after false discovery rate (Benjamini–Hochberg) correction.

Sensitivity analyses using the main subclasses of emotional disorders as outcomes (anxiety and depressive disorders) found similar association‐patterns. Of notice, anxiety symptoms at 8 years were specifically associated with anxiety disorder and showed little association with depressive disorder in adolescence (10–18 years) (Figure 2 and Table S7). In contrast, depressive symptoms at 8 years were associated with both subclasses of emotional disorders.

### 3.2 Childhood developmental trajectories of emotional and behavioral problems association with emotional disorders present in adolescence

**FIGURE 3:**
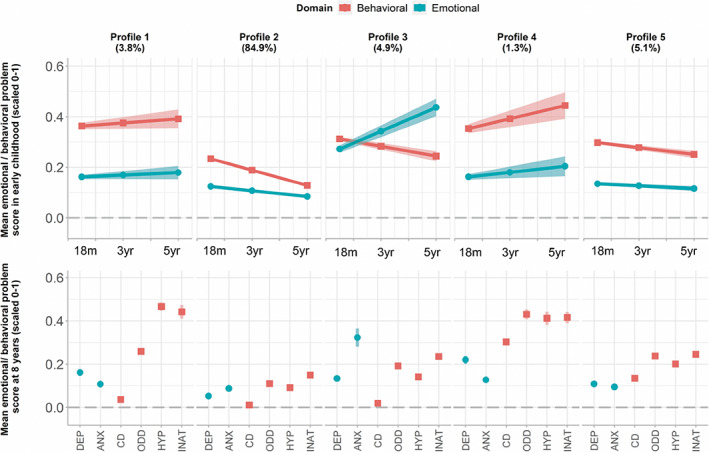
Developmental trajectories of childhood emotional and behavioral problems in early and middle childhood. Figure illustrating developmental profiles of childhood emotional and behavioral problems. Upper panel shows development of early childhood emotional (blue, circle) and behavioral (red, square) problems measured by CBCL at 18 months, 3 years and 5 years of age. Lower panel shows corresponding middle childhood mental health problems. 95% confidence‐intervals are illustrated in shaded colors. ANX, anxiety symptoms; CD, conduct disorder symptoms; DEP, depressive symptoms; HYP, hyperactivity problems; INAT, inattention problems; ODD, oppositional defiant disorder problems

Figure 3 and Table 2 summarize results for the disorder association to the developmental profiles of emotional and behavioral problems across childhood. We identified five distinct developmental profiles: Profile 1 (3.8%): Initially elevated and increasing behavioral problems followed by elevated levels of inattention and hyperactivity symptoms and moderate oppositional defiant disorder symptoms at 8 years. Profile 2 (84.9%): Initially low and decreasing early childhood emotional and behavioral problems and low symptoms at 8 years. Profile 3 (4.9%): Initially moderate and steeply increasing emotional problems followed by elevated anxiety symptoms at 8 years. Profile 4 (1.3%): Initially elevated and increasing behavioral problems along with initially low but increasing emotional problems in early childhood followed by elevated symptoms across a broad range of domains (depression, inattention, hyperactivity, oppositional defiant disorder and conduct disorder) at 8 years. Profile 5 (5.1%): Initially moderate and gradually decreasing early childhood behavioral problems with moderate levels of inattention, hyperactivity and oppositional defiant disorder symptoms at 8 years.

**TABLE 2:** Relative odds of any adolescent emotional disorder given assignment to specific developmental profile

Comparison	Odds ratio (OR)	95% CI
Profile 1 versus reference	2.15	(1.57, 2.74)
Profile 3 versus reference	3.49	(2.88, 4.10)
Profile 4 versus reference	5.00	(3.70, 6.30)
Profile 5 versus reference	1.37	(1.05, 1.69)
Profile 1 versus profile 3	0.62	(0.43, 0.81)
Profile 1 versus profile 4	0.43	(0.27, 0.60)
Profile 1 versus profile 5	1.57	(1.04, 2.11)
Profile 3 versus profile 4	0.70	(0.49, 0.90)
Profile 3 versus profile 5	2.55	(1.87, 3.22)
Profile 4 versus profile 5	3.65	(2.47, 4.83)

*Note*: Profile 2 including 84.9% (*n* = 69,522) of the individuals was used as reference class. Distribution of individuals to subsequent classes: Profile 1 (*n* = 3087, 3.77%), Profile 3 (*n* = 4045, 4.94%), Profile 4 (*n* = 1074, 1.31%), Profile 5 (*n* = 4158, 5.08%). All profiles were compared to each other. All associations were significant with *p* < 0.001. CI, confidence interval.

Individuals classified in profile 1, 3, and 4, all characterized by increasing early childhood emotional and/or behavioral problems and elevated symptoms at 8 years, were all at least twice as likely to have an adolescent diagnosis of emotional disorder as individuals in the normative class with low symptoms throughout development (Profile 1: OR: 2.15, 95% CI: 1.57–2.74, Profile 3: OR: 3.49, 95% CI: 2.88–4.10, Profile 4: OR: 5.00, 95% CI: 3.70–6.30).

Fit statistics for assessed models and demographic characteristics including distribution of adolescent emotional diagnoses for the final five developmental profiles are presented in Tables S8 and S9, respectively.

Sensitivity analyses evaluating implications of outcome definition and sex found overall the same pattern of associations in both logistic regression analyses and developmental analyses. Childhood‐onset adolescent‐persistent emotional disorders was generally stronger associated to the disorder‐related phenotypes than the main outcome of adolescent‐present emotional disorders (see Tables S10 and S11). Similarly, some disorder‐related traits (anxiety and depressive symptoms) and developmental‐profiles (profile 4) were slightly weaker associated to adolescent‐onset emotional disorders than adolescent present emotional disorders (see Tables S12 and S13). Sensitivity analyses stratifying for sex identified the same pattern in individual symptom‐relation and trajectories, however with profile 3 (dominated by emotional problems) being stronger associated with adolescent emotional disorders in males than in females (see Tables S14–S17).

## 4 DISCUSSION

The main finding of the present study was that maternal reported temperamental traits and emotional and behavioral problems in childhood are consistently and increasingly associated with diagnoses of mood and anxiety disorders in adolescence. We found consistent differences in mental traits for children later diagnosed with an emotional disorder from 6 months of age, expanding knowledge from previous studies of smaller sample size, and with shorter and/or later time of follow‐up.^20^, ^21^ Distinct trajectories of emotional and behavioral problems in childhood related to later mood or anxiety disorders in adolescence were identified. Collectively, our findings emphasize the relevance of early childhood in development of mood and anxiety disorders. From a clinical perspective, we here show that integrated information of individuals' emotional and behavioral functioning across childhood by the age of 8 years can help identify a subset of individuals with up to a 5‐fold increased risk of mood and/or anxiety disorder in adolescence.

Our findings indicate that adolescent mood and anxiety disorders are often preceded by a gradual development of emotional and behavioral problems in early childhood. Expanding knowledge from previous studies, our analysis identified differences in mental traits present from 6 months of age, increasing in degree and specificity with aging. Coherent with previous studies, trajectories of increasing symptom‐burden in childhood were related to higher risk of adolescent diagnosis in our analyses.^43^, ^44^ Sensitivity analyses finding the strongest association to childhood‐onset emotional disorders further supports mental traits potential as early signs or manifestations of disorder. Clinically our findings are of relevance for research on prevention and early identification efforts. As of today, there is no consensus regarding general screening initiatives aimed at children.^45^, ^46^ Our study indicates that divergence from typical emotional and behavioral development can be detected already in childhood. However, the study also emphasizes the complexity and relatively small effect‐sizes for signs and symptoms presenting in this age group, thus acknowledging the challenges related to identification of children with high‐risk of adolescent disorder.

Emotional dysregulation stands out as an important characteristic in the development of mood and anxiety disorders. Negative emotionality, characterized by an increased propensity to experience and react with intense negative emotions, such as sadness, anxiety, fear and anger^47^ was the temperamental trait consistently most strongly associated with later emotional disorder. Negative emotionality and emotional problems, both encompassed in the term of emotional dysregulation, are conceptualized as transdiagnostic traits related to the development and worse prognosis of mood and anxiety disorders.^48^ Association between emotional dysregulation and mood and anxiety disorders has been identified at the genetic and biological level, and the trait has been found to be a predictor of future major depression and bipolar disorder.^49^, ^50^ Our study provides novel empirical support for the importance of emotional dysregulation in the development of mood and anxiety disorders, identifying a consistent and strengthening relationship from infancy to middle childhood.

The importance of emotional dysregulation is further emphasized by the relative importance of the developmental profile 4. This profile has characteristics coherent with the controversial disruptive mood dysregulation disorder (DMDD) included in the Diagnostic and Statistical Manual of Mental disorders (DSM‐5), that is characterized by persistent irritability and recurrent temper outbursts.^51^ The combined phenotype characterizing this disorder and developmental profile, together with our findings of similar magnitude of association between emotional and behavioral problems and adolescent mood and anxiety disorders, highlight the role of dysregulation rather than emotional symptoms alone. Together this stresses the need to also consider behavioral problems as potential manifestations of emotional dysregulation or risk factors for emotional disorders, incorporating a broad developmental perspective when evaluating need for preventive interventions in this age group.

Results of our study must be interpreted while considering its limitations. Although the use of maternal reported prospective measures of childhood mental traits and clinician‐assigned diagnostic outcome avoids common rating method bias, maternal report as the only source to information on childhood mental characteristics is an important limitation of our study. The childhood characteristics captured in this study is a reflection of the maternal perception of her child, and will thus not only capture characteristics of the child but also characteristics of, and circumstances around, the mother. The group‐level bias introduced by this limitation is uncertain as influence is complex, sample and method dependent and potentially bidirectional.^52^, ^53^, ^54^, ^55^ Furthermore, it is also worth to note that prevalence of diagnosis in our sample is low compared to prevalences typically described in global epidemiologic overviews where anxiety rates in adolescence often range from 3% to 30% and depression rates 1%–4%.^14^, ^15^, ^56^ Contributing to this lower prevalence is that we, in contrary to many previous studies, only have included registry based special health care diagnosis as our outcome. In addition, some children in our analysis are still young (mean age at end of follow up is 13 years old), thus we expect additional diagnoses in the following years. Finally, despite population‐based sampling methods, the selected nature of our sample (as all birth cohorts reliant on voluntary participation) could also have biased down prevalence rates somewhat. Using clinician‐assigned diagnosis as our outcome improves validity, however it is also dependent on help‐seeking and referral to specialist health care, thus it is likely that there are undiagnosed individuals in our sample that would have been captured by a screening‐based outcome‐definition. Due to limited power, we did not test for sex‐specific associations. However, because of unclear findings of sex differences in temperament, emotional and behavioral traits,^57^ we conducted post hoc sex‐stratified sensitivity analyses finding support for generalization of our results across sex. It is also worth highlighting that this study, to our knowledge, is the first large scale Scandinavian study investigating the cross‐sectional and longitudinal relation between these early childhood mental traits and clinical diagnosis of adolescent emotional disorders. Therefore, the aim of the present study was limited to identify and describe the variables relation in the general population. To aid early identification and better understand the childhood traits' relation in development of disorder, future studies should include information from several sources and investigate the influence of genetic and environmental risk factors such as family history of emotional disorders, social support and indicators of early childhood adversity. Despite our results being in coherence with (smaller) studies in clinical samples^58^, ^59^, ^60^ our results should be replicated in a clinical sample to evaluate generalizability to clinician assessed symptoms and the full range of individuals seen in clinical settings.

## 5 CONCLUSION

Using the large, prospective population‐based MoBa pregnancy cohort with longitudinal questionnaire follow‐up and linkage to diagnoses from the nationwide patient registry, we identified specific mental traits (negative emotionality, emotional problems, and behavioral problems) in childhood that were longitudinally associated with adolescent diagnosis of mood and anxiety disorders. The disorders were further associated with specific developmental profiles of combined emotional and behavioral problems. Our profile most strongly associated with adolescent mood or anxiety disorder was coherent with symptoms of disruptive mood dysregulation disorder (DMDD). Together, this support DMDD's relevance to emotional disorders, and emphasize the importance of a broad developmental perspective when evaluating risk of developing or persisting mood and anxiety disorders. Overall, our findings support a role of emotional dysregulation in the development of mood and anxiety disorders, highlighting the importance of childhood as a formative period, and suggesting potential for prevention and early intervention initiatives.

## CONFLICT OF INTEREST

Ole A. Andreassen is consultant to HealthLytix and received a speaker's honorarium from Lundbeck and Sunovion. Ole A. Andreassen is associate editor in Acta Psychiatrica Scandinavica. Martin Tesli is field editor for genetics and translational psychiatry in Acta Psychiatrica Scandinavica. None of the remaining authors have any conflicts of interest related to this manuscript.

### PEER REVIEW

The peer review history for this article is available at https://publons.com/publon/10.1111/acps.13522.

## Supporting information


**Appendix S1:** Supporting Information.

## Data Availability

Data from the Norwegian Mother, Father and Child Cohort Study and the Medical Birth Registry of Norway used in this study are managed by the national health register holders in Norway (Norwegian Institute of public health) and can be made available to researchers, provided approval from the Regional Committees for Medical and Health Research Ethics (REC), compliance with the EU General Data Protection Regulation (GDPR) and approval from the data owners. The consent given by the participants does not open for storage of data on an individual level in repositories or journals. Researchers who want access to data sets for replication should apply through helsedata.no. Access to data sets requires approval from The Regional Committee for Medical and Health Research Ethics in Norway and an agreement with MoBa.

## References

[1] Vos T , Lim SS , Abbafati C , et al. Global burden of 369 diseases and injuries in 204 countries and territories, 1990–2019: a systematic analysis for the global burden of disease study 2019. Lancet. 2020;396(10258):1204‐1222. doi:10.1016/s0140-6736(20)30925-9 33069326 PMC7567026

[2] Beesdo‐Baum K , Knappe S . Developmental epidemiology of anxiety disorders. Child Adolesc Psychiatr Clin N Am. 2012;21(3):457‐478. doi:10.1016/j.chc.2012.05.001 22800989

[3] Rohde P , Lewinsohn PM , Klein DN , Seeley JR , Gau JM . Key characteristics of major depressive disorder occurring in childhood, adolescence, emerging adulthood, and adulthood. Clin Psychol Sci. 2013;1(1):41‐53. doi:10.1177/2167702612457599 PMC3833676 24273703

[4] Davey CG , Mcgorry PD . Early intervention for depression in young people: a blind spot in mental health care. Lancet Psychiatry. 2019;6(3):267‐272. doi:10.1016/s2215-0366(18)30292-x 30502077

[5] Arango C , Díaz‐Caneja CM , Mcgorry PD , et al. Preventive strategies for mental health. Lancet Psychiatry. 2018;5(7):591‐604. doi:10.1016/s2215-0366(18)30057-9 29773478

[6] Fusar‐Poli P , Correll CU , Arango C , Berk M , Patel V , Ioannidis JPA . Preventive psychiatry: a blueprint for improving the mental health of young people. World Psychiatry. 2021;20(2):200‐221. doi:10.1002/wps.20869 34002494 PMC8129854

[7] Hoare E , Callaly E , Berk M . Can depression Be prevented? If so, how? JAMA Psychiat. 2020;77(11):1095. doi:10.1001/jamapsychiatry.2020.1273 32579156

[8] Centers for Disease Control and Prevention . Mental health surveillance among children: United States, 2005–2011. Morbidity and Mortality Weekly Report Supplement/Vol. 62/No. 2; 2013.

[9] Fox SE , Levitt P , Nelson Iii CA . How the timing and quality of early experiences influence the development of brain architecture. Child Dev. 2010;81(1):28‐40. doi:10.1111/j.1467-8624.2009.01380.x 20331653 PMC2846084

[10] Malik F , Marwaha R . Developmental Stages of Social Emotional Development in Children. StatPearls. StatPearls Publishing; 2022. https://www.ncbi.nlm.nih.gov/books/NBK534819/. 30521240

[11] Creswell C , Waite P , Cooper PJ . Assessment and management of anxiety disorders in children and adolescents. Arch Dis Child. 2014;99(7):674‐678. doi:10.1136/archdischild-2013-303768 24636957 PMC4078705

[12] Petito A , Pop TL , Namazova‐Baranova L , et al. The burden of depression in adolescents and the importance of early recognition. J Pediatr. 2020;218:265‐267.e261. doi:10.1016/j.jpeds.2019.12.003 31932020

[13] Andershed A‐K , Andershed H . Risk and protective factors among preschool children: integrating research and practice. J Evidence Informed Social Work. 2015;12(4):412‐424. doi:10.1080/15433714.2013.866062 25794349

[14] Bennett SW , John T . Epidemiology, pathogenesis, clinical manifestations, and course. *UptoDate* (Literature review current through: May 2022. This topic last updated: May 12, 2022. ed.); 2022.

[15] Bonin L . Pediatric unipolar depression: epidemiology, clinical features, assessment, and diagnosis. In *UpToDate* (Literature review current through: May 2021. This topic last updated: Apr 19, 2021. ed.); 2021.

[16] Ka L , R E , W. K , J. G , Lje B . Associations between facets and aspects of big five personality and affective disorders:a systematic review and best evidence synthesis. J Affect Disord. 2021;288:175‐188. doi:10.1016/j.jad.2021.03.061 33901698

[17] Kotov R , Gamez W , Schmidt F , Watson D . Linking “big” personality traits to anxiety, depressive, and substance use disorders: a meta‐analysis. Psychol Bull. 2010;136(5):768‐821. doi:10.1037/a0020327 20804236

[18] Mulraney M , Coghill D , Bishop C , et al. A systematic review of the persistence of childhood mental health problems into adulthood. Neurosci Biobehav Rev. 2021;129:182‐205. doi:10.1016/j.neubiorev.2021.07.030 34363845

[19] Zarrella I , Russolillo LA , Caviglia G , Perrella R . Continuity and discontinuity between psychopathology of childhood and adulthood: a review on retrospective and prospective studies. Res Psychother. 2017;20(2):101‐109. doi:10.4081/ripppo.2017.248 PMC7451328 32913738

[20] Shore L , Toumbourou JW , Lewis AJ , Kremer P . Review: longitudinal trajectories of child and adolescent depressive symptoms and their predictors: a systematic review and meta‐analysis. Child Adolesc Mental Health. 2018;23(2):107‐120. doi:10.1111/camh.12220 32677332

[21] Nandi A , Beard JR , Galea S . Epidemiologic heterogeneity of common mood and anxiety disorders over the lifecourse in the general population: a systematic review. BMC Psychiatry. 2009;9(1):31. doi:10.1186/1471-244x-9-31 19486530 PMC2700109

[22] Bongers IL , Koot HM , van der Ende J , Verhulst FC . Developmental trajectories of externalizing behaviors in childhood and adolescence. Child Dev. 2004;75(5):1523‐1537. doi:10.1111/j.1467-8624.2004.00755.x 15369529

[23] Magnus P , Birke C , Vejrup K , et al. Cohort profile update: the Norwegian mother and child cohort study (MoBa). Int J Epidemiol. 2016;45(2):382‐388. doi:10.1093/ije/dyw029 27063603

[24] Magnus P , Irgens LM , Haug K , Nystad W , Skjærven R , Stoltenberg C . Cohort profile: the Norwegian mother and child cohort study (MoBa). Int J Epidemiol. 2006;35(5):1146‐1150. doi:10.1093/ije/dyl170 16926217

[25] Bates JE , Freeland CAB , Lounsbury ML . Measurement of infant difficultness. Child Dev. 1979;50(3):794‐803. doi:10.2307/1128946 498854

[26] Mathiesen KS , Tambs K . The EAS temperament questionnaire‐factor structure, age trends, reliability, and stability in a Norwegian sample. J Child Psychol Psychiatry. 1999;40(3):431‐439. doi:10.1111/1469-7610.00460 10190344

[27] Vollrath ME , Hampson SE , Torgersen S . Constructing a short form of the hierarchical personality inventory for children (HiPIC): the HiPIC‐30. Pers Mental Health. 2016;10(2):152‐165. doi:10.1002/pmh.1334 27120426

[28] Buss AH , Plomin R . Temperament (PLE: Emotion): Early Developing Personality Traits. revised ed. Psychology Press; 2014.

[29] Achenbach TM , Ruffle TM . The child behavior checklist and related forms for assessing behavioral/emotional problems and competencies. Pediatr Rev. 2000;21(8):265‐271. 10922023 10.1542/pir.21-8-265

[30] Angold A , Costello EJ , Messer SC , Pickles A . Development of a short questionnaire for use in epidemiological studies of depression in children and adolescents. Int J Methods Psychiatr Res. 1995;5(4):237‐249.

[31] Birmaher B , Brent DA , Chiappetta L , Bridge J , Monga S , Baugher M . Psychometric properties of the screen for child anxiety related emotional disorders (SCARED): a replication study. J Am Acad Child Adolesc Psychiatry. 1999;38(10):1230‐1236. doi:10.1097/00004583-199910000-00011 10517055

[32] Silva RR , Alpert M , Pouget E , et al. A rating scale for disruptive behavior disorders, based on the DSM‐IV item Pool. Psychiatr Quart. 2005;76(4):327‐339. doi:10.1007/s11126-005-4966-x 16217627

[33] World Health Organization , ed. ICD‐10: international statistical classification of diseases and related health problems: tenth revision. 2nd ed. World Health Organization; 2004.

[34] Bullis JR , Boettcher H , Sauer‐Zavala S , Farchione TJ , Barlow DH . What is an emotional disorder? A transdiagnostic mechanistic definition with implications for assessment, treatment, and prevention. Clin Psychol Sci Pract. 2019;26(2):e12278. doi:10.1037/h0101755

[35] Long JA . Jtools: analysis and presentation of social scientific data. R Package Version 2.1.0; 2020.

[36] Zeileis A . Econometric computing with HC and HAC covariance matrix estimators. J Stat Softw. 2004;11(10):1‐17. doi:10.18637/jss.v011.i10

[37] Benjamini Y , Hochberg Y . Controlling the false discovery rate: a practical and powerful approach to multiple testing. J R Stat Soc B Methodol. 1995;57(1):289‐300. doi:10.1111/j.2517-6161.1995.tb02031.x

[38] Hannigan LJ , Askeland RB , Ask H , et al. Genetic liability for schizophrenia and childhood psychopathology in the general population. Schizophr Bull. 2021;47(4):1179‐1189. doi:10.1093/schbul/sbaa193 33561255 PMC8266611

[39] Nylund‐Gibson K , Grimm RP , Masyn KE . Prediction from latent classes: a demonstration of different approaches to include distal outcomes in mixture models. Struct Equ Model Multidiscip J. 2019;26(6):967‐985. doi:10.1080/10705511.2019.1590146

[40] Vermunt JK . Latent class modeling with covariates: two improved three‐step approaches. Political Anal. 2010;18(4):450‐469. doi:10.1093/pan/mpq025

[41] Petersen KJ , Qualter P , Humphrey N . The application of latent class analysis for investigating population child mental health: a systematic review. Front Psychol. 2019;10:1214. doi:10.3389/fpsyg.2019.01214 PMC6548989 31191405

[42] Muthén LK , Muthén BO . Mplus user's guide. 8th ed. Muthén & Muthén; 1998‐2017.

[43] Battaglia M , Garon‐Carrier G , Côté SM , et al. Early childhood trajectories of separation anxiety: bearing on mental health, academic achievement, and physical health from mid‐childhood to preadolescence. Depress Anxiety. 2017;34(10):918‐927. doi:10.1002/da.22674 28833904

[44] Dekker MC , Ferdinand RF , van Lang NDJ , Bongers IL , van der Ende J , Verhulst FC . Developmental trajectories of depressive symptoms from early childhood to late adolescence: gender differences and adult outcome. J Child Psychol Psychiatry Allied Discip. 2007;48(7):657‐666. doi:10.1111/j.1469-7610.2007.01742.x 17593146

[45] Siu AL . Screening for depression in children and adolescents: US preventive services task force recommendation Statement. Pediatrics. 2016;137(3):e20154467. doi:10.1542/peds.2015-4467 26908686

[46] Walter HJ , Bukstein OG , Abright AR , et al. Clinical practice guideline for the assessment and treatment of children and adolescents with anxiety disorders. J Am Acad Child Adolesc Psychiatry. 2020;59(10):1107‐1124. doi:10.1016/j.jaac.2020.05.005 32439401

[47] Kann SJ , O'Rawe JF , Huang AS , Klein DN , Leung H‐C . Preschool negative emotionality predicts activity and connectivity of the fusiform face area and amygdala in later childhood. Soc Cogn Affect Neurosci. 2017;12(9):1511‐1519. doi:10.1093/scan/nsx079 28992271 PMC5737644

[48] Dougherty LR , Smith VC , Bufferd SJ , et al. Preschool irritability: longitudinal associations with psychiatric disorders at age 6 and parental psychopathology. J Am Acad Child Adolesc Psychiatry. 2013;52(12):1304‐1313. doi:10.1016/j.jaac.2013.09.007 24290463 PMC3860177

[49] Kostyrka‐Allchorne K , Wass SV , Sonuga‐Barke EJS . Research review: do parent ratings of infant negative emotionality and self‐regulation predict psychopathology in childhood and adolescence? A systematic review and meta‐analysis of prospective longitudinal studies. J Child Psychol Psychiatry. 2020;61(4):401‐416. doi:10.1111/jcpp.13144 31696514

[50] Vidal‐Ribas P , Brotman MA , Valdivieso I , Leibenluft E , Stringaris A . The status of irritability in psychiatry: a conceptual and quantitative review. J Am Acad Child Adolesc Psychiatry. 2016;55(7):556‐570. doi:10.1016/j.jaac.2016.04.014 27343883 PMC4927461

[51] Rao U . DSM‐5: disruptive mood dysregulation disorder. Asian J Psychiatr. 2014;11:119‐123. doi:10.1016/j.ajp.2014.03.002 25453714 PMC4254488

[52] Kristensen S , Henriksen TB , Bilenberg N . The child behavior checklist for ages 1.5–5 (CBCL/1½–5): assessment and analysis of parent‐ and caregiver‐reported problems in a population‐based sample of Danish preschool children. Nord J Psychiatry. 2010;64(3):203‐209. 20085433 10.3109/08039480903456595

[53] Najman JM , Williams GM , Nikles J , et al. Bias influencing maternal reports of child behaviour and emotional state. Soc Psychiatry Psychiatr Epidemiol. 2001;36(4):186‐194. 11518032 10.1007/s001270170062

[54] Olino TM , Michelini G , Mennies RJ , Kotov R , Klein DN . Does maternal psychopathology bias reports of offspring symptoms? A study using moderated non‐linear factor analysis. J Child Psychol Psychiatry. 2021;62(10):1195‐1201. 33638150 10.1111/jcpp.13394

[55] Roubinov DS , Epel ES , Adler NE , Laraia BA , Bush NR . Transactions between maternal and child depressive symptoms emerge early in life. J Clin Child Adolesc Psychol. 2022;51(1):61‐72. 31453717 10.1080/15374416.2019.1644649 PMC7044043

[56] World Health Organization . Adolescent mental health. WHO; 2021. https://www-who-int..no/news-room/fact-sheets/detail/adolescent-mental-health.

[57] Chaplin TM , Aldao A . Gender differences in emotion expression in children: a meta‐analytic review. Psychol Bull. 2013;139(4):735‐765. doi:10.1037/a0030737 23231534 PMC3597769

[58] Eaton C , Tarver J , Shirazi A , et al. A systematic review of the behaviours associated with depression in people with severe–profound intellectual disability. J Intellect Disabil Res. 2021;65(3):211‐229. 33426741 10.1111/jir.12807

[59] Eyre O , Langley K , Stringaris A , Leibenluft E , Collishaw S , Thapar A . Irritability in ADHD: associations with depression liability. J Affect Disord. 2017;215:281‐287. 28363151 10.1016/j.jad.2017.03.050 PMC5409953

[60] Oakley B , Loth E , Murphy DG . Autism and mood disorders. Int Rev Psychiatry. 2021;33(3):280‐299. 33648430 10.1080/09540261.2021.1872506

